# Aging Induces Changes in the Somatic Nerve and Postsynaptic Component without Any Alterations in Skeletal Muscles Morphology and Capacity to Carry Load of *Wistar* Rats

**DOI:** 10.3389/fnins.2017.00688

**Published:** 2017-12-18

**Authors:** Walter Krause Neto, Wellington de Assis Silva, Adriano P. Ciena, Romeu R. de Souza, Carlos A. Anaruma, Eliane F. Gama

**Affiliations:** ^1^Laboratory of Morphoquantitative Studies and Immunohistochemistry, Department of Physical Education, São Judas Tadeu University, São Paulo, Brazil; ^2^Laboratory of Morphology and Physical Activity, Department of Physical Education, São Paulo State University, Rio Claro, Brazil

**Keywords:** brain aging, frailty, neurodegeneration, sarcopenia

## Abstract

The present study aimed to analyze the morphology of the peripheral nerve, postsynaptic compartment, skeletal muscles and weight-bearing capacity of *Wistar* rats at specific ages. Twenty rats were divided into groups: 10 months-old (ADULT) and 24 months-old (OLD). After euthanasia, we prepared and analyzed the tibial nerve using transmission electron microscopy and the soleus and plantaris muscles for cytofluorescence and histochemistry. For the comparison of the results between groups we used dependent and independent Student's *t-*test with level of significance set at *p* ≤ 0.05. For the tibial nerve, the OLD group presented the following alterations compared to the ADULT group: larger area and diameter of both myelinated fibers and axons, smaller area occupied by myelinated and unmyelinated axons, lower numerical density of myelinated fibers, and fewer myelinated fibers with normal morphology. Both aged soleus and plantaris end-plate showed greater total perimeter, stained perimeter, total area and stained area compared to ADULT group (*p* < 0.05). Yet, aged soleus end-plate presented greater dispersion than ADULT samples (*p* < 0.05). For the morphology of soleus and plantaris muscles, density of the interstitial volume was greater in the OLD group (*p* < 0.05). No statistical difference was found between groups in the weight-bearing tests. The results of the present study demonstrated that the aging process induces changes in the peripheral nerve and postsynaptic compartment without any change in skeletal muscles and ability to carry load in *Wistar* rats.

## Introduction

Aging is a natural phenomenon associated with structural, functional, biochemical, molecular, and genetic alterations in many cells and tissues (Verdú et al., 1996; Ceballos et al., 1999; Shokouhi et al., 2008; Shen et al., 2011; Ibebunjo et al., 2012; Marzetti et al., 2013; Moldovan et al., 2016; Pannérec et al., 2016; Klaips et al., 2017). Several changes in the peripheral nervous system (PNS), normally related to the process of advancing age, have already been reported such as: regional sarcopenia, loss of neuromuscular junction stability, altered synaptic transmission and function, impaired motor unit recruitment, and impaired local genes control (Mortelliti et al., 1990; Hinman et al., 2006; Wang et al., 2007; Deschenes et al., 2011; Nishimune et al., 2012; Cheng et al., 2013; Tamaki et al., 2014; Pannérec et al., 2016). In humans, the motor unit (MU) population declines at later ages and it is accompanied by a reduction in the number and diameter of nerve fibers in the ventral root (Lexell, 1997). Evidence suggests that alterations such as motorneuron degeneration, functional denervation, accompanied by structural and functionality changes in the neuromuscular junction (NMJ), and loss of motor units contribute to the progression of muscular aging process in rodents and humans (Deschenes, 2011; Jang and Van Remmen, 2011; Gonzalez-Freire et al., 2014; Tudoraşcu et al., 2014; Hepple and Rice, 2016; Piasecki et al., 2016; Rygiel et al., 2016; Gilmore et al., 2017).

The decrease in the number of myofibers observed in many skeletal muscles is directly linked with a loss of motoneurons and predominance of functional denervation (Deschenes et al., 2010; Deschenes, 2011; Tamaki et al., 2014; Pannérec et al., 2016). In fact, although numerous mechanisms are proposed to explain age-related muscle fiber loss, perhaps the most reasonable is that peripheral denervation is caused by the loss of motoneurons and NMJ remodeling (Vandervoort, 2002; Deschenes et al., 2010; Pannérec et al., 2016). Such a process is proposed to occur in humans and several lineages of laboratory rodents. Nevertheless, the diversity of lineages and ages studied leaves a major doubt as to whether all these proposed changes occur at the same time in a specific rodent lineage. For example, while evidence presents changes at postsynaptic NMJ sites of Fisher 344 rats (Deschenes et al., 2010), others have failed (Deschenes et al., 2007, 2016). Further, Scheib and Höke (2016) have found that Schwann cells and macrophages have their reduced immunological ability to induce axonal regeneration as early as 18 months-old in Brown-Norway rats. Shokouhi et al. (2008) demonstrated that sciatic nerve from *Wistar* rats presents increased lipid peroxidation, apoptosis of Schwann cells and axon/myelin ultrastructural alterations at 15 months of age. Interesting, major disorganization and marked nerve fiber loss only occur after 20 months of age in the Swiss lineage (Ceballos et al., 1999). Being more critical, the very definition of nomenclatures regarding the animal's age as an adult, middle-aged, aged, old, and very old may vary according to the rodent's lineage and species (Andreollo et al., 2012; Dutta and Sengupta, 2016). According to Pannérec et al. (2016), as expected for muscle phenotype in hindlimbs, *Wistar* rats might be divided under the following categories: adult (8–10 months-old), early-sarcopenic (18–20 months-old), and sarcopenic (22–24 months-old).

The morphology of the neuromuscular system is determined to be entropically organized. Thus, each movement, whether simple or complex, is the consequence of a highly precise pattern of the activity of motoneurons controlled by supraspinal motor centers, associated with the biomechanical properties of the muscular system (Rekling et al., 2000; Godde and Voelcker-Rehage, 2017; Karmali et al., 2017). Movement disorders are among of the most frequent changes in neurological functions seen in the elderly (Verdú et al., 2000). Aging is associated with a simultaneous decline in the quality of sensory information provided to the brain and a deterioration in motor control (Karmali et al., 2017). Yet, reduced physical activity seen during elderly may alter brain activation, impairing motor status and increasing motor deficits (Godde and Voelcker-Rehage, 2017). Functional deficits may be the consequence of a loss of nerve fibers (Knox et al., 1989; Hashizume and Kanda, 1995; Ugrenovic et al., 2016), myelin sheath abnormalities (Ceballos et al., 1999), changes in extracellular matrix (Esquisatto et al., 2014), as well as changes in neuronal or glial expression of membrane channels, neurotrophic factors, and cell adhesion molecules, thereby decreasing axoplasmic transport (Milde et al., 2015; Krishman et al., 2016; Luo et al., 2016; Moldovan et al., 2016; Takagishi et al., 2016). Although many mechanisms seeking explanations for functional deficits have already been suggested, it is not known whether the weight-bearing capacity of *Wistar* rats declines with advancing age. Moreover, it is not possible to affirm that ultrastructural alterations in the peripheral nerve cause reduction of the physical function during the aging.

Sarcopenia is defined as the multi-factorial outcome associated with reduction of muscle mass and strength levels, thus decreasing physical-functional capacity (Jang and Van Remmen, 2011; Tamaki et al., 2014; Pannérec et al., 2016). In the specific literature, muscle strength was defined as the ability of the neuromuscular system to generate sufficient muscle strength to overcome, sustain or yield to a given external load or resistance. Classically, old human beings lose part of their functional capacity, affecting directly daily life activities. In the experimental area, functional capacity is studied evaluating muscles in an isolated way (Degens and Alway, 2003) instead of evaluating the animal's voluntary capacity to generate muscular tension associated to a physical activity similar to that done by humans. In this way, to evaluate the capacity of voluntary muscular force in a physical activity similar to that practice by humans, but able to be executed by rodents would become necessary (Seo et al., 2014).

Due to the great variability of results found in the literature, a great question remains to be answered regarding the neuromuscular changes expected to occur in the peripheral nerve ultrastructure, and skeletal muscles and neuromuscular junction morphology and physical capacity of *Wistar* rats at specific ages. Thus, the present study aimed to analyze the morphology of the peripheral nerve, postsynaptic compartment, skeletal muscles, and weight-bearing capacity of adult and old *Wistar* rats.

## Methods

This study was authorized by the Committee on Ethics in Animal Use (CEAU - Protocol 001/2013) of the St. Jude Tadeu University (USJT). Twenty male *Wistar* rats provided by the USJT bioterium were divided into groups: ADULT- 10 months-old (*n* = 10) and OLD- 24 months-old (*n* = 10). Several studies presented evidence that these ages is representative of the adult and old phases of *Wistar* and others rats lineages (Deschenes et al., 2000, 2011, 2013a,b, 2016; Pannérec et al., 2016). At 24 months, changes in the neuromuscular system are expected to have occurred (Ceballos et al., 1999; Deschenes et al., 2010, 2012; Pannérec et al., 2016). As our purpose is to replicate frequently used methodologies and evaluate if the facts are confirmed within the *Wistar* lineage, the ages used in this study are in agreement with the one proposed by our objective. Also, many studies presented already neuromuscular alterations in male (Hashizume and Kanda, 1995; Jeronimo et al., 2008; Shokouhi et al., 2008, Deschenes et al., 2013b; Tamaki et al., 2014; Pannérec et al., 2016; Sakita et al., 2016) and female rodents (Verdú et al., 1996; Ceballos et al., 1999; Chai et al., 2011; Cheng et al., 2013). These data allowed us to include only male mattes.

The animals were housed in polypropylene boxes (a maximum of three animals each) provided with controlled ambient conditions of temperature (22°C) and illumination (12 h light and 12 dark hours). For all groups were provide commercial reference food for rats and water *ad libitum*.

### Euthanasia and tissue analysis

The animals were euthanized by CO_2_ inhalation method. After this procedure, we removed the tibial nerve and soleus (SL) and plantaris (PL) muscles, preparing them for morpho-quantitative analysis techniques. We chose the tibial nerve by innervating the SL and PL muscles. The SL and PL muscles were chosen according to their predominance of muscle fiber typology (slow/oxidative and fast/glycolytic, respectively).

### Transmission electronic microscopy

After an incision, a fragment of ~0.5 cm in length from the tibial nerve was removed from the posterior portion of the right leg. Then, we fixed the nerve fragment in 2.5% glutaraldehyde solution in phosphate buffer (0.2 M, pH 7.3) for 3 h (Pianca et al., 2015; Carbone et al., 2017; Krause Neto et al., 2017b). Subsequently, we washed the material three times with the same buffer solution for 5 min at a time. The material was placed in a solution of 1% osmium tetroxide in phosphate buffer for 2 h. The fragments remained overnight in 0.5% uranyl acetate. In the morning we washed the material with the plug, dehydrated in increasing series of alcohol and propylene oxide for 8 h, under rotation. The nerves were included in pure resin (Spurr) for cross-section. The material remained in this stage for 5 h and then left in the same resin at 60°C for a further 3 days period. After completion of the material preparation procedure, semi-thin sections were done, the histological slides prepared and the tissue stained with toluidine blue (Ceballos et al., 1999; Pianca et al., 2015). After selection of the fields in the semi-thin sections, the ultra-thin sections were obtained with a diamond knife, in ultramicrotome (Sorvall MT-2), and contrasted with uranyl acetate and lead citrate, finally being analyzed by the transmission electron microscope. The techniques employed here, were in agreement with other publications done by our research group (Pianca et al., 2015; Carbone et al., 2017; Krause Neto et al., 2017b).

The material was taken to the electron transmission microscope (Jeol JSM1010, ICB, USP) and images were made using a magnification of 1,500x and 3,000x for stereological and morphometric analysis, respectively. Preparation of the material and images were done by the Department of Anatomy and Laboratory of Electronic Microscopy of the Institute of Biomedical Sciences of the University of São Paulo (ICB-USP).

**Figure 1:**
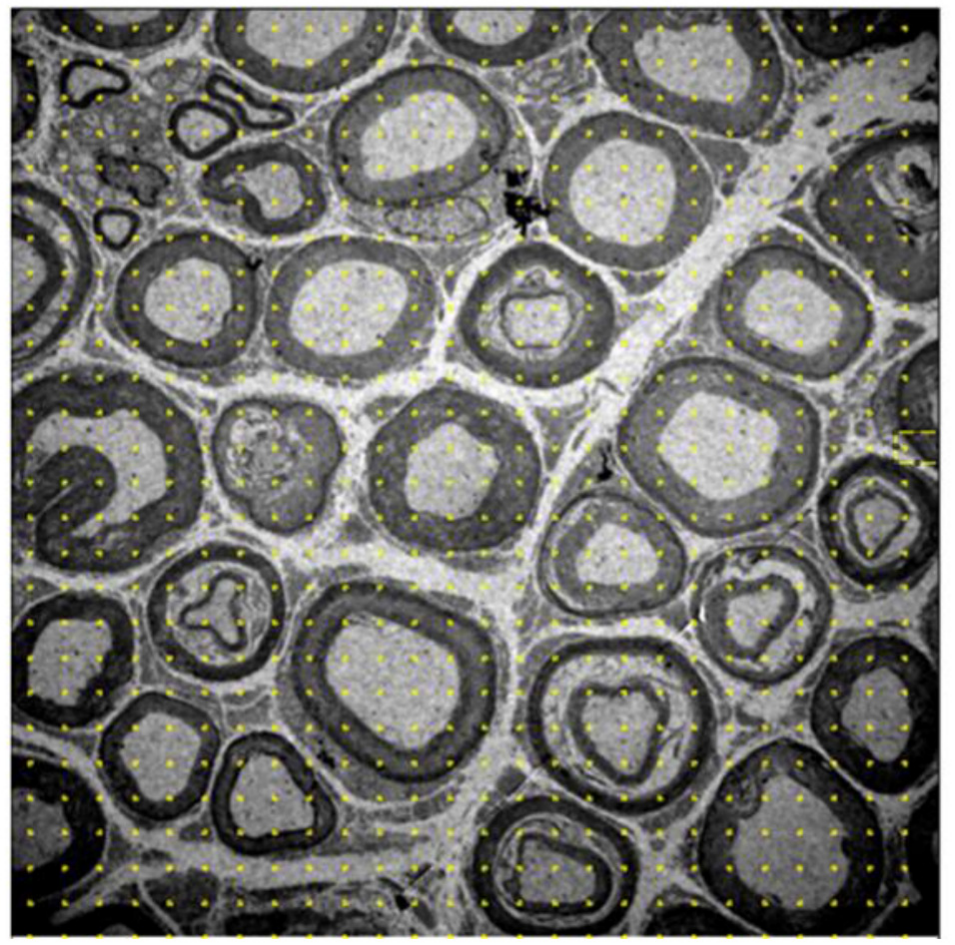
Image of the 10-month-old *Wistar* rat tibial nerve, representative of the stereology technique for acquisition of volume density of myelinated, nonmyelinated and interstitial spaces. The grid of points is placed on the image, whose representativity is transformed into percentages of occupation for each cell in question. Increase of 1,500x.

**Figure 2:**
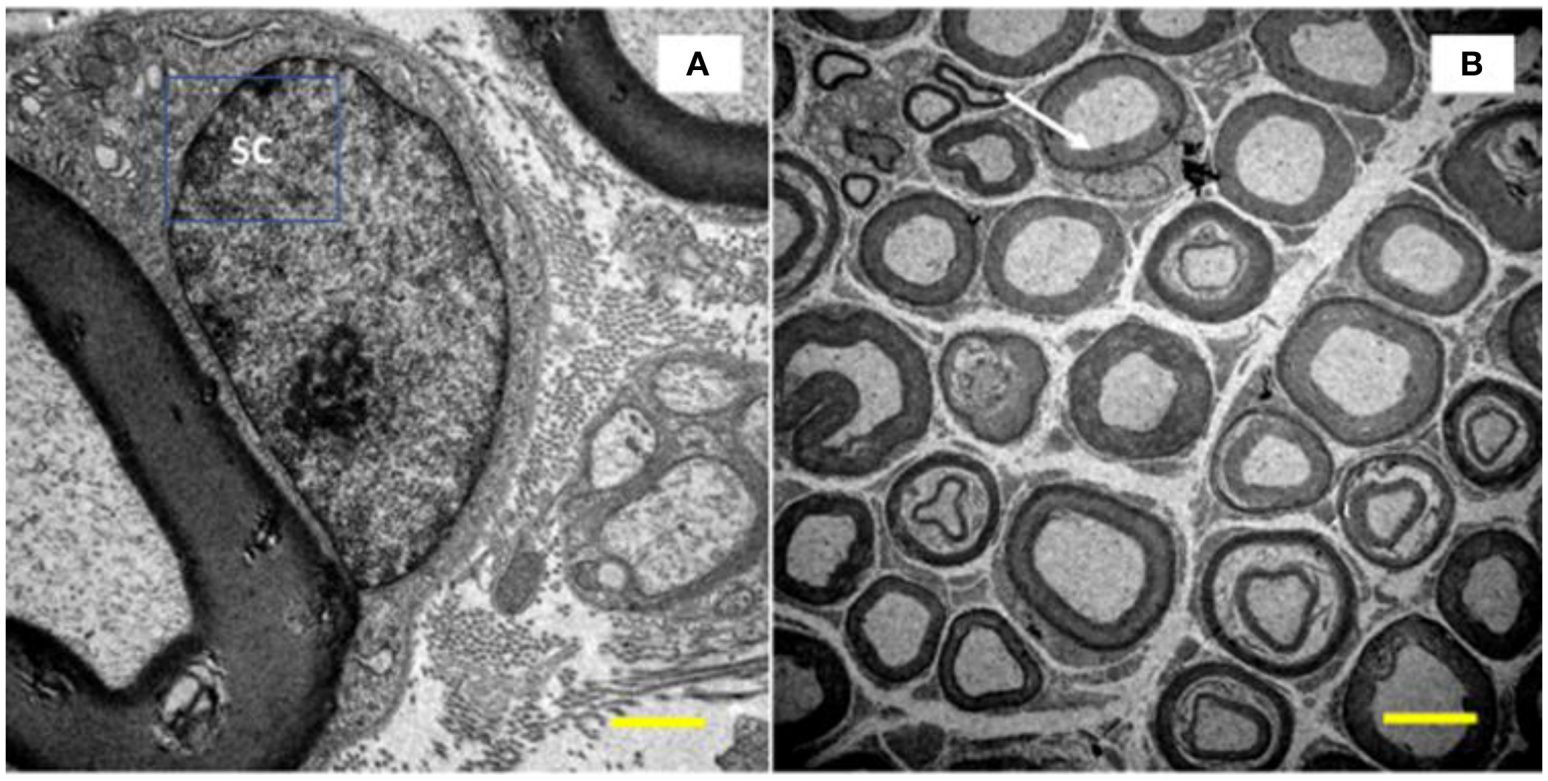
Image of the tibial nerve of 10 months-old *Wistar* rats. On the left side, a Schwann cell (SC) located peripherally the myelin sheath of its axon (**A**, increase of 10,000x) was visualized. In **(B)**, at the top of the image (white arrow), it is show a myelinated axon with its SC at the nucleus level (increase of 1,500x).

For stereology, we captured 25 images from each animal/group with final magnification of 1,500x. The amount of images captured was sufficient to assess at least 15% of all nerve (Ceballos et al., 1999). This same strategy was performed for other quantifications. Volume and numerical density were quantified as described below:

- Volume density: myelinated fibers, unmyelinated axons, and interstitium. A system with 588 points was placed on each image and points that fell on each of the previously mentioned structures were quantified, and the data were automatically transformed into percentages relative to the total number of points (Figure 1).
- Numerical density: myelinated fibers, unmyelinated axons, and Schwann cells at the nuclei level. The structures were quantified, excluding those that touched the lower and left edges of the image. We also calculated the ratio of unmyelinated axons by myelinated fibers (UA/MF), through the quotient between the number of unmyelinated axons and myelinated fibers. The numerical density of Schwann cells was determined from those contained at the periphery of the myelinated axons and at the nuclei level (Figure 2). In order to measure and evaluate these parameters, we used the software Image J.

Both stereological techniques are used in our laboratory to quantify various outcomes in nerves and other tissues (Pianca et al., 2015; Krause Neto and Gama, 2017, and Krause Neto et al., 2017b).

**Figure 3:**
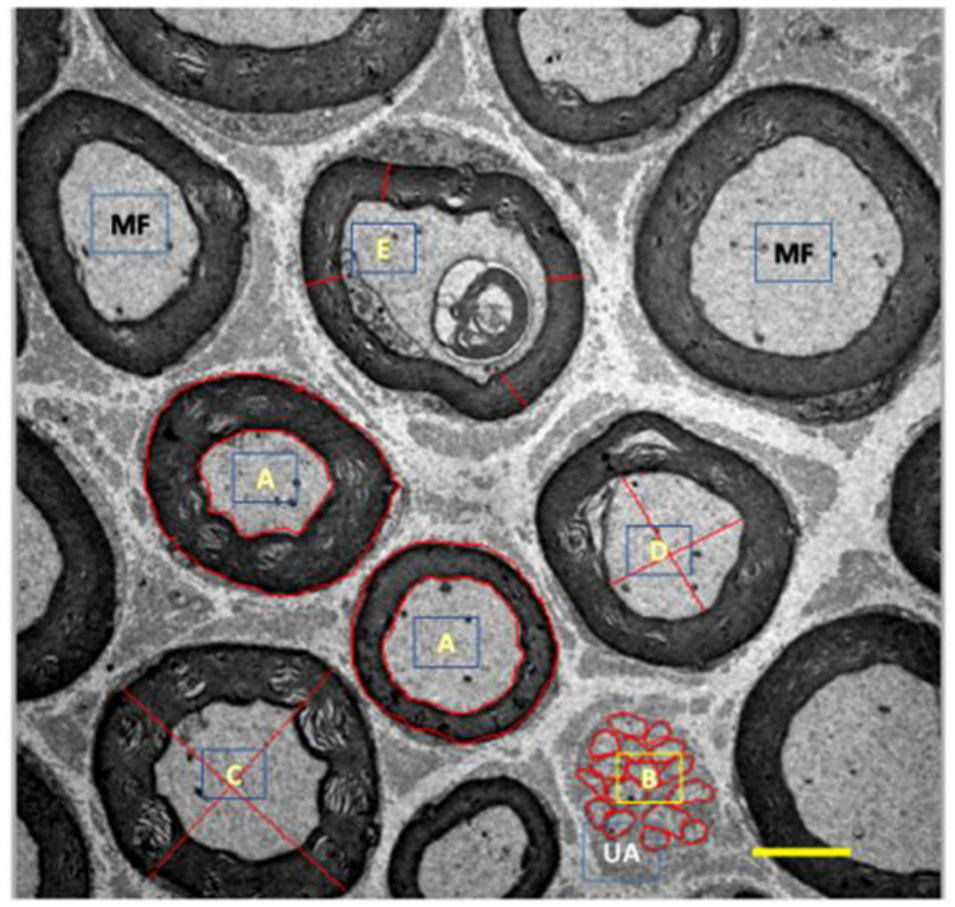
Illustration of myelinated (MF, black) and unmyelinated (UA, white) fibers from tibial nerve of 10 months-old *Wistar* rats. A, represents area drawn around the myelin sheath and myelinated axon; B, represents area drawn around the unmyelinated axons; C, represents cross-shaped strokes to calculate the mean diameter of the myelinated fiber; D, represents the cross-shaped tracing for calculating the mean diameter of the myelinated axon; E, represents the tracings for the calculation of the mean thickness of the myelin sheath. 3,000x magnification.

For morphometry, we captured 35 images of each rodent from each group (allowed us to measure over 300 myelinated fibers per group) using a 3,000x magnification and quantified: cross-sectional area of myelinated fibers (μm^2^), myelinated (μm^2^), and unmyelinated (μm^2^) axons, mean diameter (μm) of myelinated fibers and axons, mean thickness of myelin sheath (μm), and G ratio. The mean diameters were calculated from the mean between largest and smallest diameters of each structure. To calculate the G ratio, we used the quotient between the diameter of the axon and the myelinated fiber. Finally, the mean thickness of the myelin sheath was determined by the average of four equivalent cross-shaped traces on the image. For this, we use the Axiovision 4.8 program. Figure 3 presents measurement tools used here.

**Figure 4:**
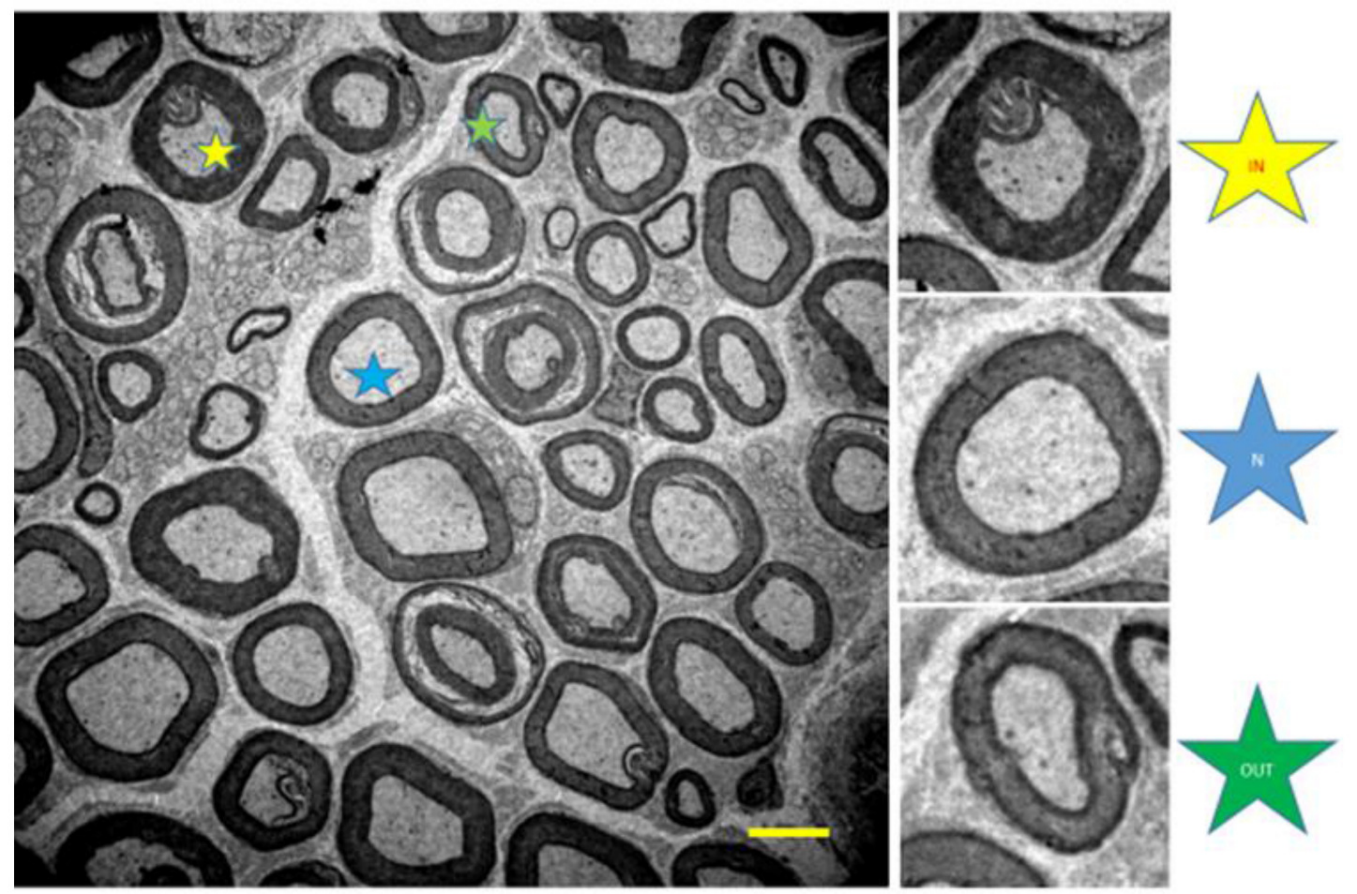
Illustration of the tibial nerve of 10 months-old *Wistar* rats. Normal myelinated fibers (N, blue star) and with internal changes (IN, yellow star) and external (OUT, green star). 1,500x magnification.

A qualitative analysis (qualitative stereology) of myelinated axons was performed quantifying the total number of axons, axons with normal morphology (N), and fibers with internal (IN), and external (OUT) changes (see Figure 4; Ceballos et al., 1999).

### Cytofluorescence

For this study, we used the Inverted Microscope (LSM780-NLO Zeiss) from the Headquarters Laboratory of the National Institute of Photonics Applied to Cell Biology of the State University of Campinas (INFABIC-UNICAMP). The preparation of the slides took place in the Laboratory of Morphology and Physical Activity of the Paulista State University “Júlio de Mesquita Filho” (UNESP-Rio Claro).

Soon after the extraction and cleaning of the SL and PL muscles, we removed the middle third of each muscle, leaving them in nitrogen liquid and then kept in a cold tank at −80°C. In order to visualize the postsynaptic component of each NMJ, we made longitudinal cuts of 100 μm thickness, using the cryotome at −20°C. To avoid tissue contraction, the slides were pretreated in 3% ethylenediaminetetraacetic acid solution. Sections were washed six times for 5 min each in phosphate-buffered saline (PBS) containing 1% bovine serum albumin (BSA). Then the sections were incubated overnight in a humidified chamber at 4°C in a solution containing α-bungarotoxin conjugated rhodamine (BTX; Molecular Probes, Eugene, OR-T-1175), diluted 1:600 in PBS. This antibody has the function of marking the acetylcholine receptors and consequently enables the analysis of the end-plate. After this period, the sections were given a final wash (6 × 5 min) before being lightly coated with Prolong (Molecular Probes, Eugene, OR-P10144) and having their coverslips applied. The slides were then encoded with respect to the intervention group in order to allow assessment of the morphology of the end-plate. Thereafter, they were stored at −20°C until analysis.

**Figure 5:**
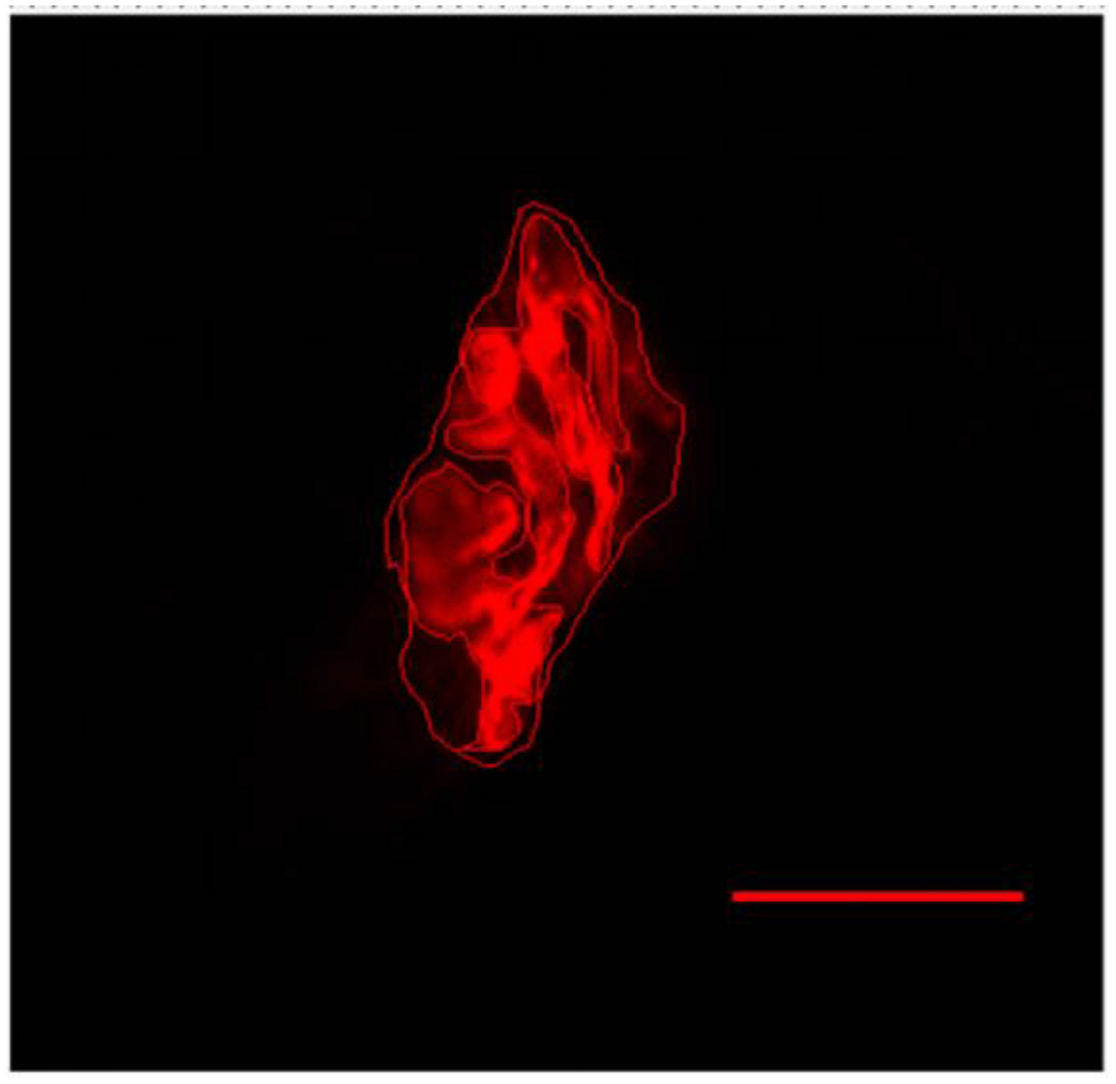
Representative image of the postsynaptic compartment of plantaris muscle of 10 months-old *Wistar* rats. Red labeling of acetylcholine (ACh) receptors with α-bungarotoxin conjugated rhodamine. Manually drawn lines for determination of the total perimeter, or the length covering the entire end-plate composed of groups of stained receptors and non-stained regions interspersed within these groups, stained perimeter, or composite length, which includes the recipients stained with uncorrected regions interspersed between groups of recipients, the stained area, or the areas occupied by cumulative groups of recipients ACh. Increase by 1,000x.

For this study 12–15 NMJ images of each muscle/animal/groups were captured, containing an end-plate, with final magnification of 1,000x (Deschenes et al., 2011). In the analysis of the postsynaptic compound of the NMJ we measured the total perimeter (μm), or the length covering the entire end-plate composed of groups of stained receptors and non-stained regions interspersed within these groups, stained perimeter (μm), or composite length, which includes the recipients stained with uncorrected regions interspersed between groups of recipients, the stained area (μm^2^), or the areas occupied by cumulative groups of recipients ACh, and the dispersion of the end-plates, which was evaluated by dividing the stained area of the end-plate by its total area and multiplying the value by 100 (Deschenes et al., 2013b) (please see Deschenes et al., 2016; Figure 5). To do this, we use the software Axiovision 4.8.

### Histochemistry

After sectioning the tissue in the thickness of 8 μm, the sections were incubated for 30 min at 37°C in a solution containing 10 mg of ATP dissolved in 2 drops of distilled water added with 10 ml of glycine/NaCl buffer CaCl_2_ and hitting the pH to 9.4 added with DDT. Then, we washed the cuts in distilled water and incubated for 2 min in 2% cobalt chloride for 3 times. Again, we wash in distilled water and dehydrate in ascending alcohol series (70, 90, 95, and 100%) and brighten in xylol. Finally we set up in Balsamo of Canada. For the method at pH 4.3 and 4.6, we preincubated the cuts in 0.1 M sodium acetate buffer with 10 mM EDTA for 10 min at 4°C at pH 4.3 and 4.6. Tissues were washed with distilled water and incubate for 2 min in 2% cobalt chloride for 3 times. Again, we washed in distilled water and dehydrated in series of increasing alcohol (70, 90, 95, and 100%), lightened in xylol. Finally we set up in Canada balsam.

To determine the muscular volume density (Vv), the slides were photographed under a light microscope with a final magnification of 100x. For this analysis, 30 photographs were taken and calculated the Vv of type I, IIa, IIx/b, and interstitium fibers of each muscle/animal/group. Also, we counted the ratio of nuclei per muscle fiber based on the quotient between the number of nuclei and muscle fibers. In these procedures, we used the Image J software.

**Figure 6:**
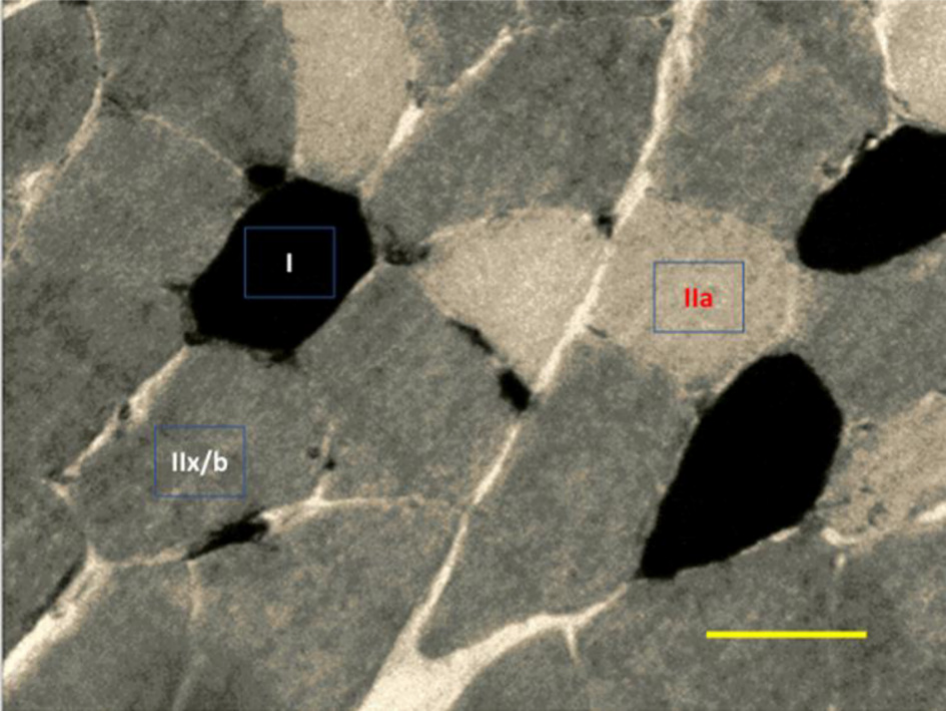
Representative image of ATPase myosin labeling for determination of muscle fiber types. Darkest myofibers are type I, lightest myofibers are type IIa, and intermediately stained myofibers are type II x/b. 400x magnification.

For morphometry, 30 photographs of each muscle/animal/group with final magnification of 400x were analyzed. In each slide it was possible to measure the cross-sectional area (μm^2^) of muscle fibers type I, IIa, and IIx/b (Figure 6). For this procedure, we use software Axiovision 4.8.

### Ladder climbing test

The ladder climbing test was applied to evaluate whether the amount of load carried, on a voluntary basis, would be different between adult and old animals (Krause Neto et al., 2017a). Knowing that the physical activity performed in the daily life has characteristics of strength resistance, we applied the test in a stair climbing model, whose model approximates daily activities performed by humans. Further, this procedure is characteristic of the manifestation of maximum force resistance by requiring the animal to scale the equipment as often as possible from a pre-determined load (see Krause Neto et al., 2017b).

**Figure 7:**
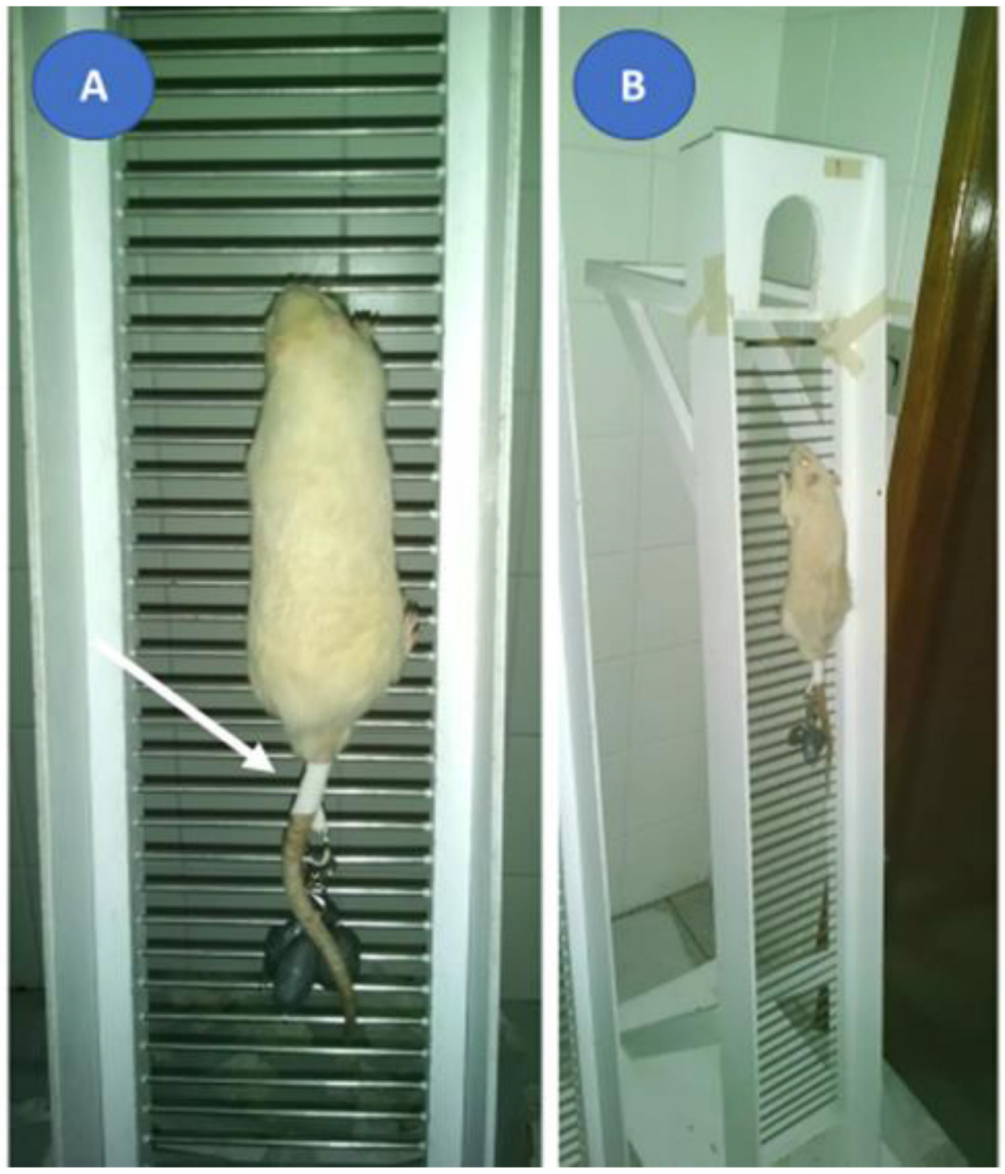
Ladder climbing equipment proposed by Hornberger and Farrar (2004). A demonstrative image of the rodent climbing with a tether attached to the proximal portion of the animal's tail (**A**, white arrow). A demonstration image of all the equipment and the climbing route that the rodent carried out **(B)**. Each animal should climb the ladder, from the base of the equipment to the top.

For this procedure, each rodent went through the process of familiarization with ladder climbing equipment. During adaptation period, each rodent climbed three times the ladder, separated by 60 s, starting from distinct positions (upper third, middle, and base) during 5 consecutive days. The animals had no help or reward to climb the equipment. In the following week all rodents performed the maximum loaded carrying test (MLCT) on two familiarization sessions (S1 vs. S2). Lead loads were attached to the proximal portion of the rodent's tail. Each animal had to climb from the base to the top of the climbing equipment (Figure 7). The option to perform the initial tests in two sessions was motivated by the existence of evidence, which demonstrated that there is a need for more than one sedentary load test session, thus reducing the initial strength gain bias supported by the improvement of the coordinative pattern (Dias et al., 2005). This data was documented in humans of different ages and levels of trainability, however it is not known if this fact is also possible to occur in experiments with laboratory rodents. After 72 h of S1, the rodents underwent the same procedures, characterizing the second moment (S2). The two tests sessions were applied using the same methodology and the same load progressions.

The MLCT consisted on the animal climbing the ladder as often as possible, from 50% of their body weight, adding a new percentage (10%) to each successful attempt. The test was conducted until the animal was unable to climb the ladder for at least two consecutive trials. Between each attempt, the animal had an interval of 2 min. The tests were applied in a simple-blind fashion. The test scientist responsible did not know which group was being tested. In the end, we quantified the number of climbs and the maximum load loaded in each MLCT session.

### Statistical analysis

Data are presented by mean and standard deviation. For the comparison of the results from the same group Student's *t-*test was calculated for dependent samples (S1 vs. S2). To compare groups, we used *t-*test for independent samples. For the statistical calculations we used SPSS software version 21.0 and set the level of significance at *p* ≤ 0.05. Values of *p* between 0.051 and 0.07 were considered as statistical trend.

## Results

### Tibial nerve

#### Morphometry

**Figure 8:**
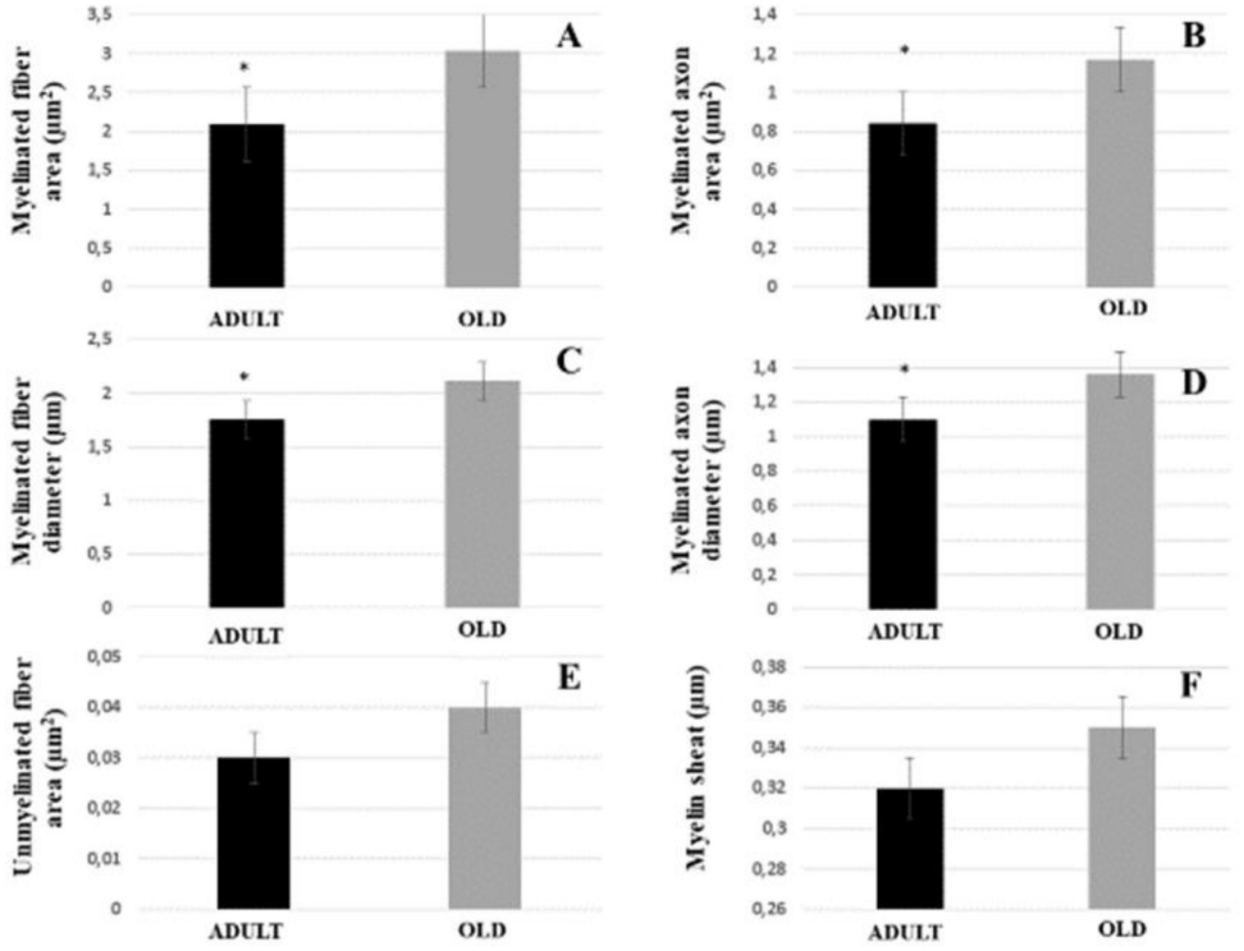
Morphometric parameters of the tibial nerve of Wistar rats between ADULT (black bar) and OLD (gray bar) groups. Myelinated fiber area and diameter **(A,C)**, myelinated axon area and diameter **(B,D)**, unmyelinated fiber area **(E)** and myelin sheath thickness **(F)**. ^*^Represents a significant difference between groups (*p* < 0.05).

The area and diameter of both myelinated fibers and axons increased significantly in the OLD group (*p* < 0.05). Area of the unmyelinated axons and myelin sheath were not different between the groups (Figure 8). Yet, G ratio was not different between groups (0.63 ± 0.03 e 0.64 ± 0.03, respectively for ADULT and OLD groups).

#### Stereology

**Figure 9:**
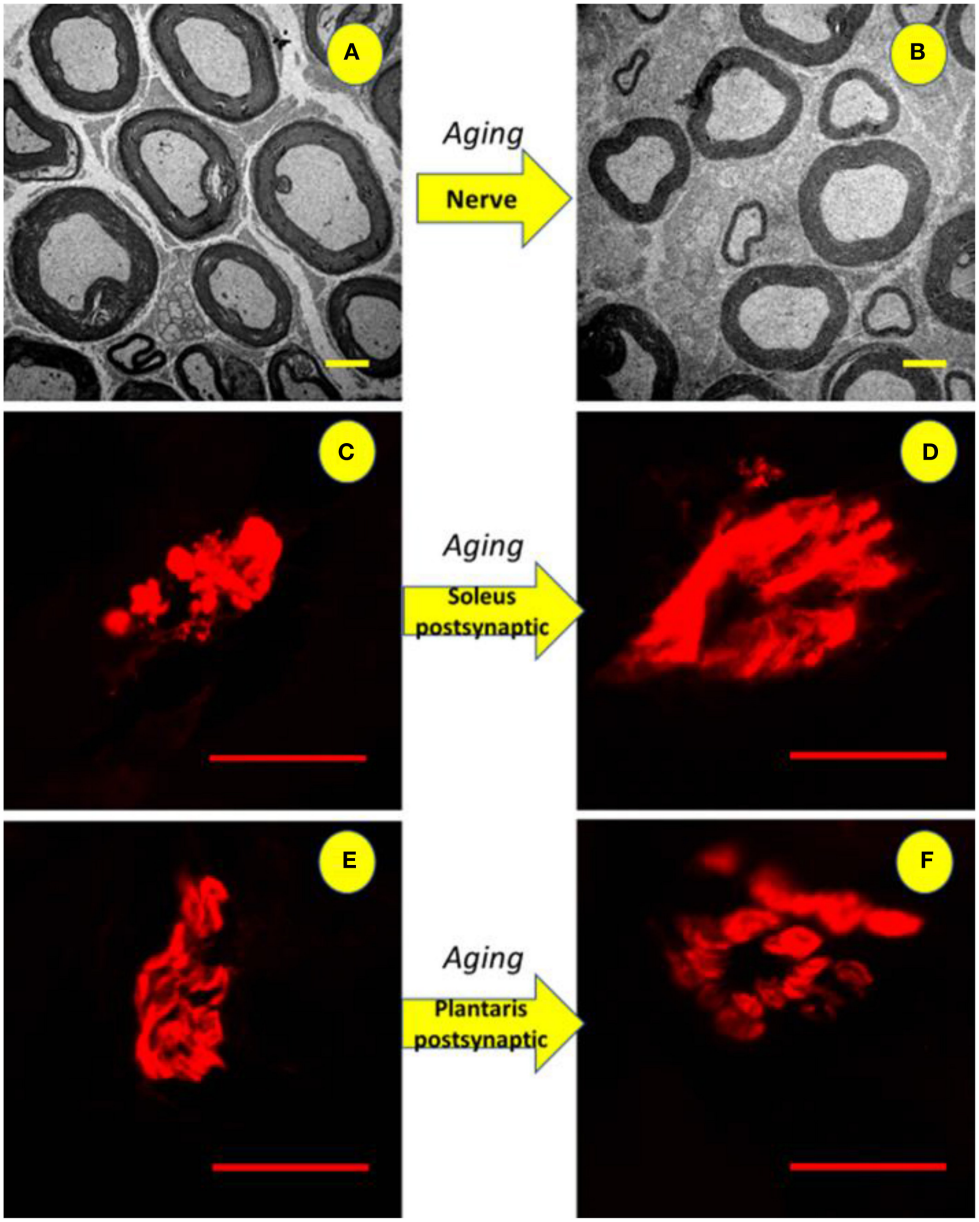
Illustration of the tibial nerve and postsynaptic compartment morphology of 10 months-old (ADULT, left) and 24 months-old (OLD, right) *Wistar* rats. Upper panels **(A,B)** show reduction in the number and space occupied by myelinated axons (3,000x magnification) with increased interstitial space. For soleus **(C,D)** and plantaris **(E,F)** show enlargement of end-plates caused by age advance (1,000x magnification).

Data are shown in Table 1. The area occupied by myelinated and unmyelinated axons decreased significantly in the OLD group (*p* < 0.05). Interstitial space increased with aging (*p* < 0.05; Figures 9A,B). The numerical density of myelinated fibers decreased statistically in the OLD group (*p* < 0.05). There was no significant difference for the unmyelinated axon and UA/MF ratio between groups.

**Table 1:** Tibial nerve stereology of *Wistar* rats from ADULT (10 months-old) and OLD (24 months-old) groups.

	**ADULT (*n* = 10)**	**OLD (*n* = 10)**	***P*-value**
**STEREOLOGY**			
Myelinated axon (%)	64.42 ± 5.11	58.69 ± 5.01	0.025
Unmyelinated axon (%)	4.8 ± 2.11	1.93 ± 1.76	0.005
Interstitium (%)	30.77 ± 5.48	39.39 ± 5.06	0.002
Myelinated fiber (*n*)	11.71 ± 2.34	7.73 ± 3.26	0.001
Unmyelinated axon (*n*)	18.57 ± 12.23	12.2 ± 9.85	0.133
UA/MF ratio (*n*)	1.51 ± 0.91	1.67 ± 1.48	0.736
Schwann cell nuclei (*n*)	0.5 ± 0.52	0.11 ± 0.33	0.075
**QUALITY STEREOLOGY**			
Total myelinated fibers (*n*)	38.9 ± 8.35	32.78 ± 4.36	0.062
Normal myelinated fibers (*n*)	32.3 ± 7.06	24.33 ± 2.74	0.006
(In) myelinated fibers (*n*)	9.9 ± 2.76	8.67 ± 3.08	0.371
(Out) myelinated fibers (*n*)	2.5 ± 1.71	2.22 ± 0.83	0.665

*Values are presented as mean ± standard deviation. Vv, volume density (%); Nv, numerical density (number); UA/MF ratio, unmyelinated axon/myelinated fiber*.

The OLD group tended to have less total myelinated fibers than ADULT (*p* = 0.062). The number of myelinated fibers with normal morphology was lower in the OLD group compared to the ADULT group (*p* < 0.05). For the other outcomes, there was no significant difference between groups.

#### Postsynaptic compartment

Figure 9 shows enlarged area of ACh receptors in both skeletal muscles from OLD group (Soleo, C and D; plantaris, E and F).

**Figure 10:**
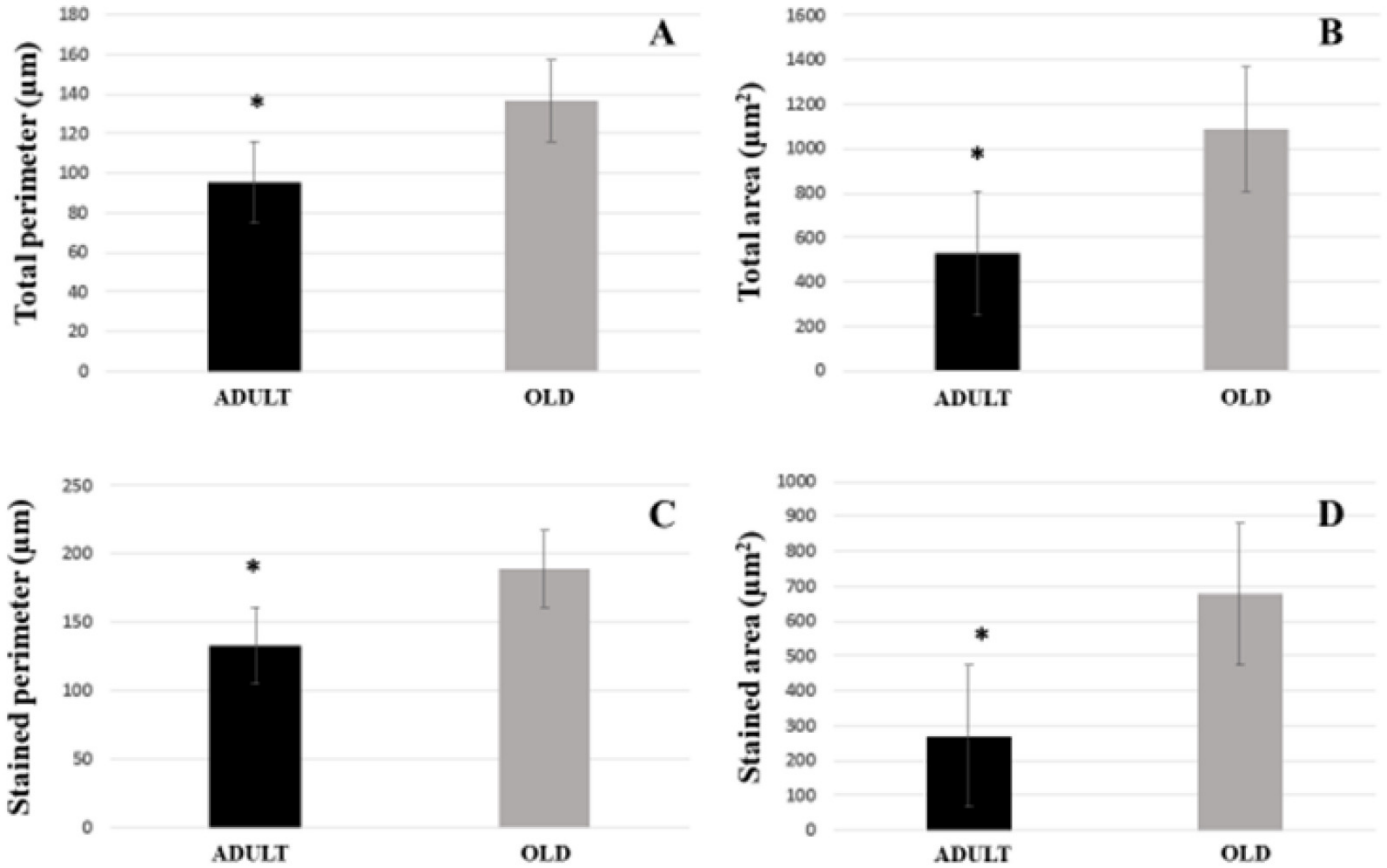
Graphic of postsynaptic components [total perimeter **(A)**, total area **(B)**, stained perimeter **(C)**, and stained area **(D)**] of the soleus muscle of Wistar rats from the adult (10 months-old) and the old (24 months-old) groups. Total perimeter, or the length covering the entire end-plate composed of groups of stained receptors and non-stained regions interspersed within these groups, stained perimeter, or composite length, which includes the recipients stained with uncorrected regions interspersed between groups of recipients, the stained area, or areas occupied by cumulative groups of recipients ACh. ^*^Represents a significant difference between groups (*p* < 0.05).

**Figure 11:**
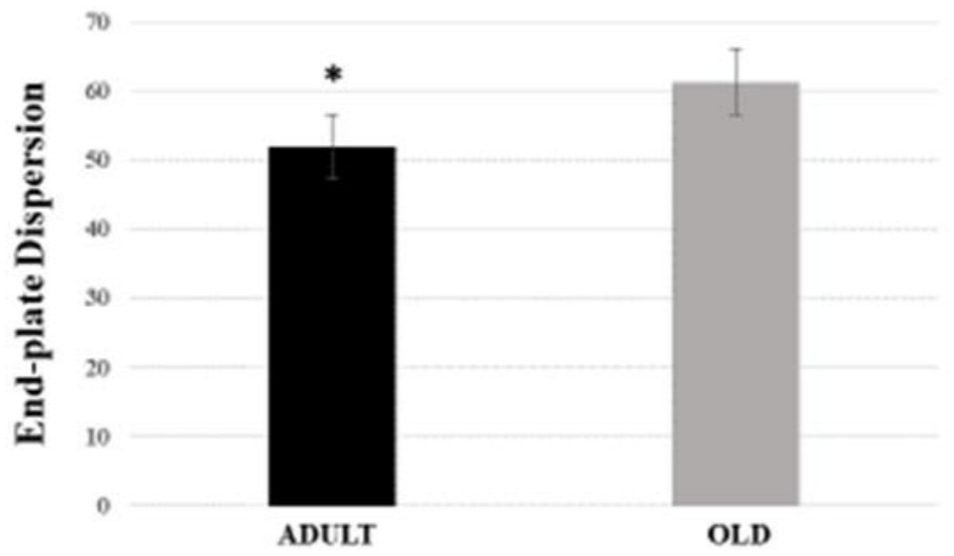
End-plate dispersion of the soleus muscle of Wistar rats from adult (10 months-old) and old (24 months-old) groups. Dispersion was evaluated by dividing the stained area of the end-plate by it total area and multiplying the value by 100. ^*^Represents a significant difference between the groups (*p* < 0.05).

For Soleus, total perimeter, stained perimeter, total area and stained area were higher in the OLD group compared to ADULT (Figure 10, *p* < 0.05). Also, end-plate dispersion were larger in OLD compared to ADULT (Figure 11, *p* < 0.05).

**Figure 12:**
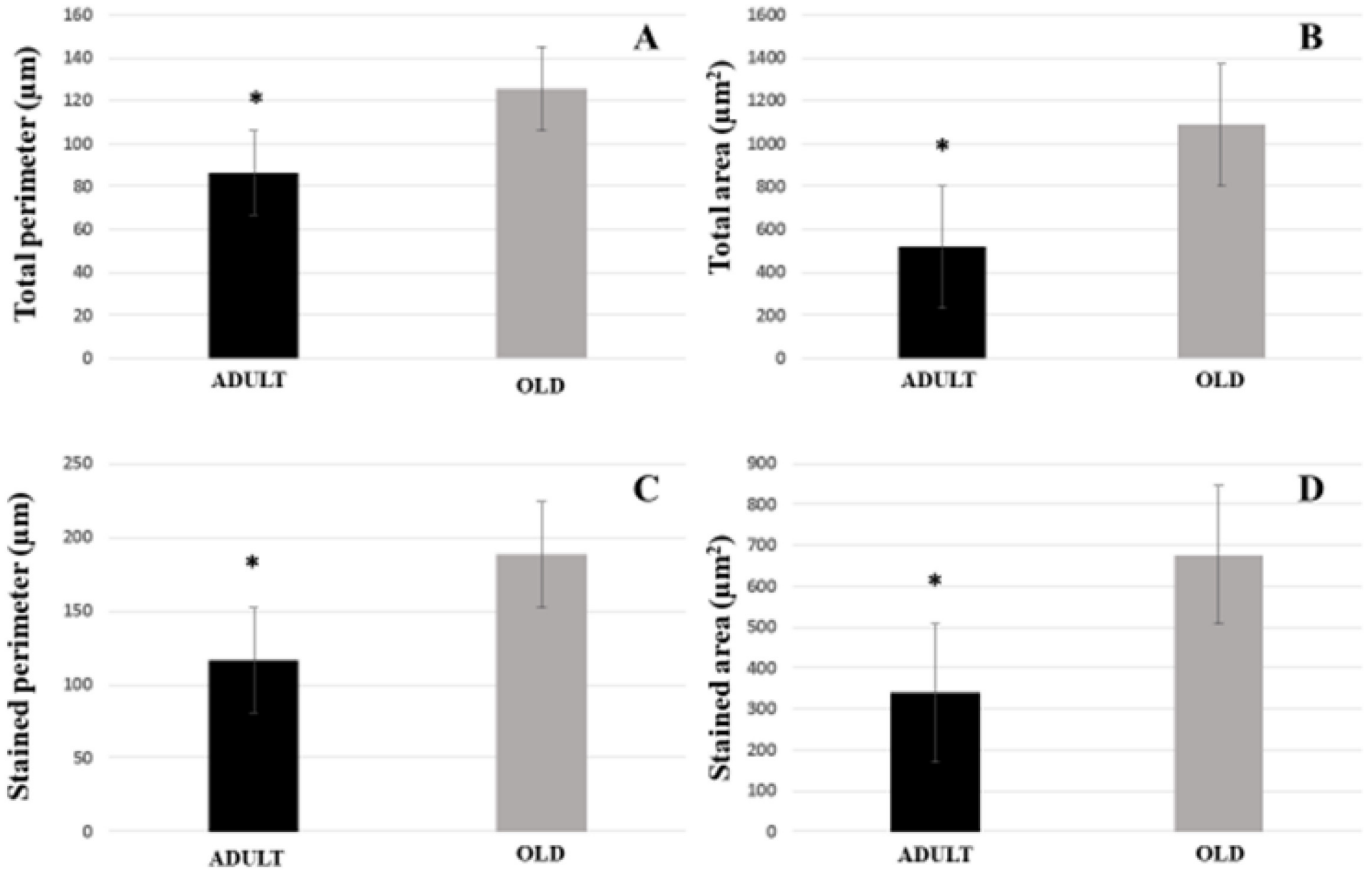
Postsynaptic components [total perimeter **(A)**, total area **(B)**, stained perimeter **(C)**, and stained area **(D)**] of the plantaris muscle of Wistar rats from the adult (10 months-old) and old (24 months-old) groups. Total perimeter, or the length covering the entire end-plate composed of groups of stained receptors and non-stained regions interspersed within these groups, stained perimeter, or composite length, which includes the recipients stained with uncorrected regions interspersed between groups of recipients, the stained area, or areas occupied by cumulative groups of recipients ACh. ^*^Represents a significant difference between groups (*p* < 0.05).

**Figure 13:**
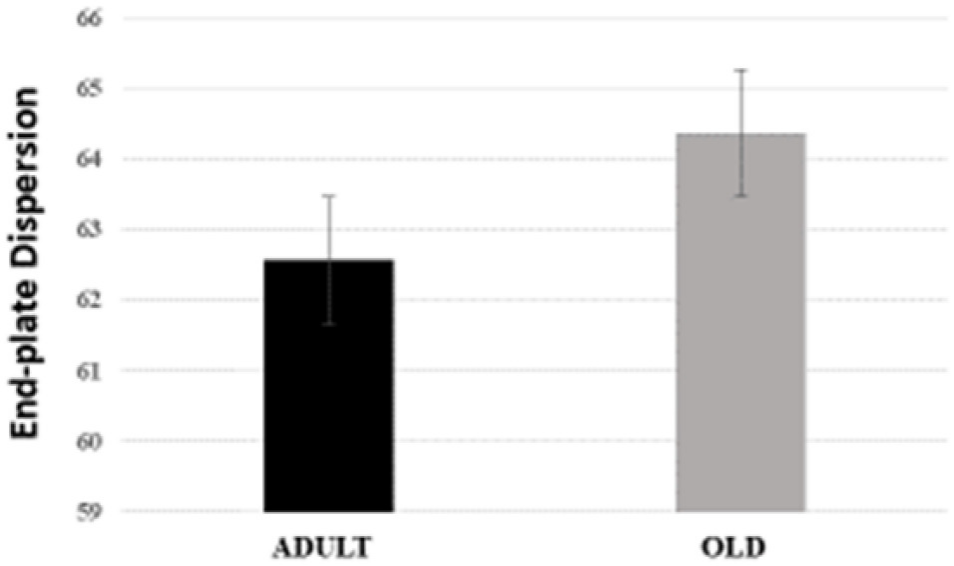
End-plate dispersion of plantaris muscle of Wistar rats from adult (10 months-old) and old (24 months-old) groups. Dispersion was evaluated by dividing the stained area of the end-plate by it total area and multiplying the value by 100.

For plantaris end-plate total perimeter, stained perimeter, total area, and stained area were higher in the OLD group compared to ADULT (*p* < 0.05) (Figure 12). However, the dispersion of the end plate was not different between the groups (Figure 13).

#### Skeletal muscles

**Figure 14:**
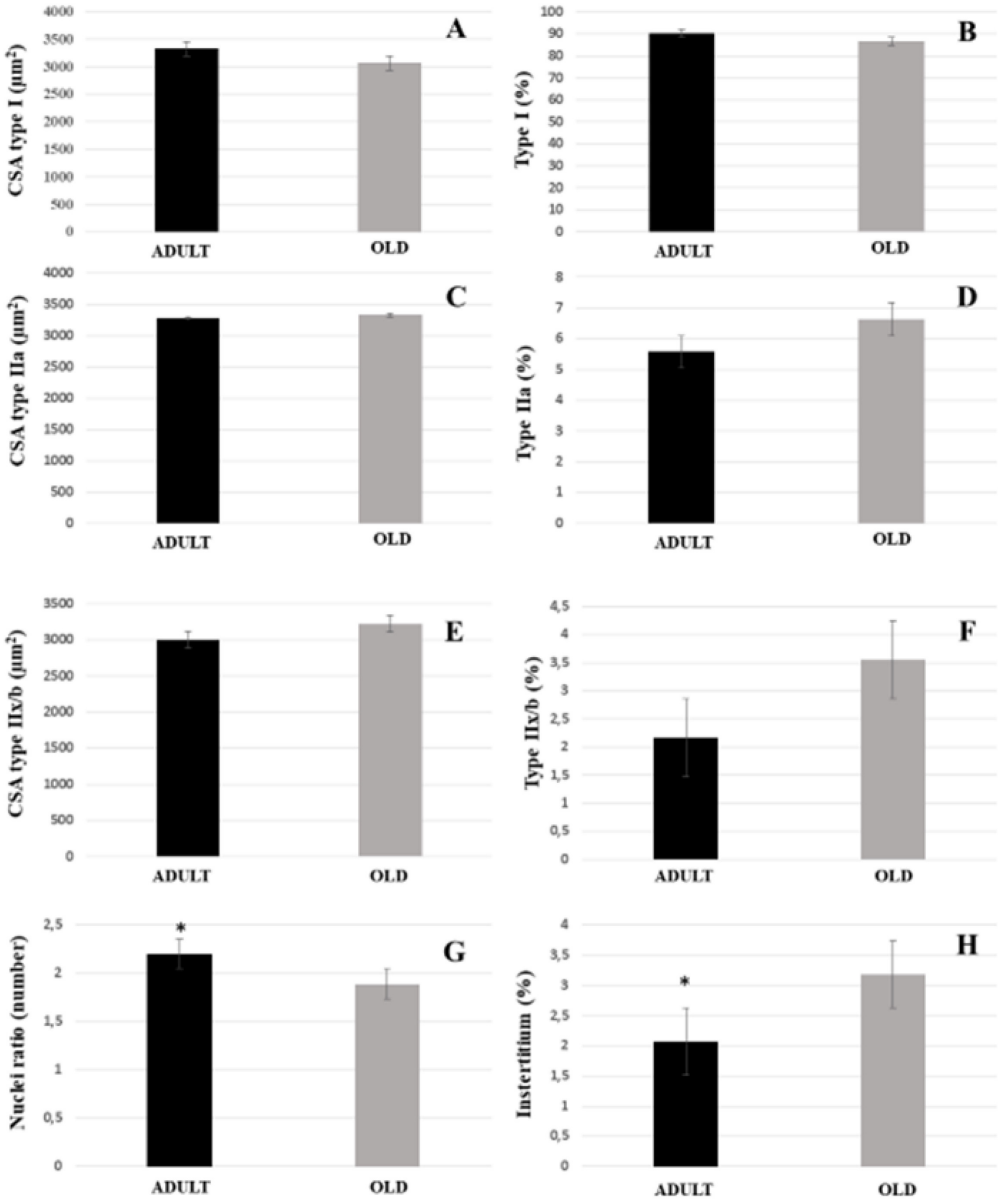
Morphoquantitative parameters of soleus muscle from adult (10 months-old) and old (24 months-old) Wistar rats. Cross-sectional area of type I **(A)**, IIa **(C)**, and IIx/b **(E)**. Stereology of type I **(B)**, IIa **(D)**, IIx/b **(F)**, and interstitium **(H)**. Nuclei ratio **(G)** was calculated dividing number of nuclei in each field by total number of muscle fibers. ^*^Represents a significant difference between the groups (*p* < 0.05).

**Figure 15:**
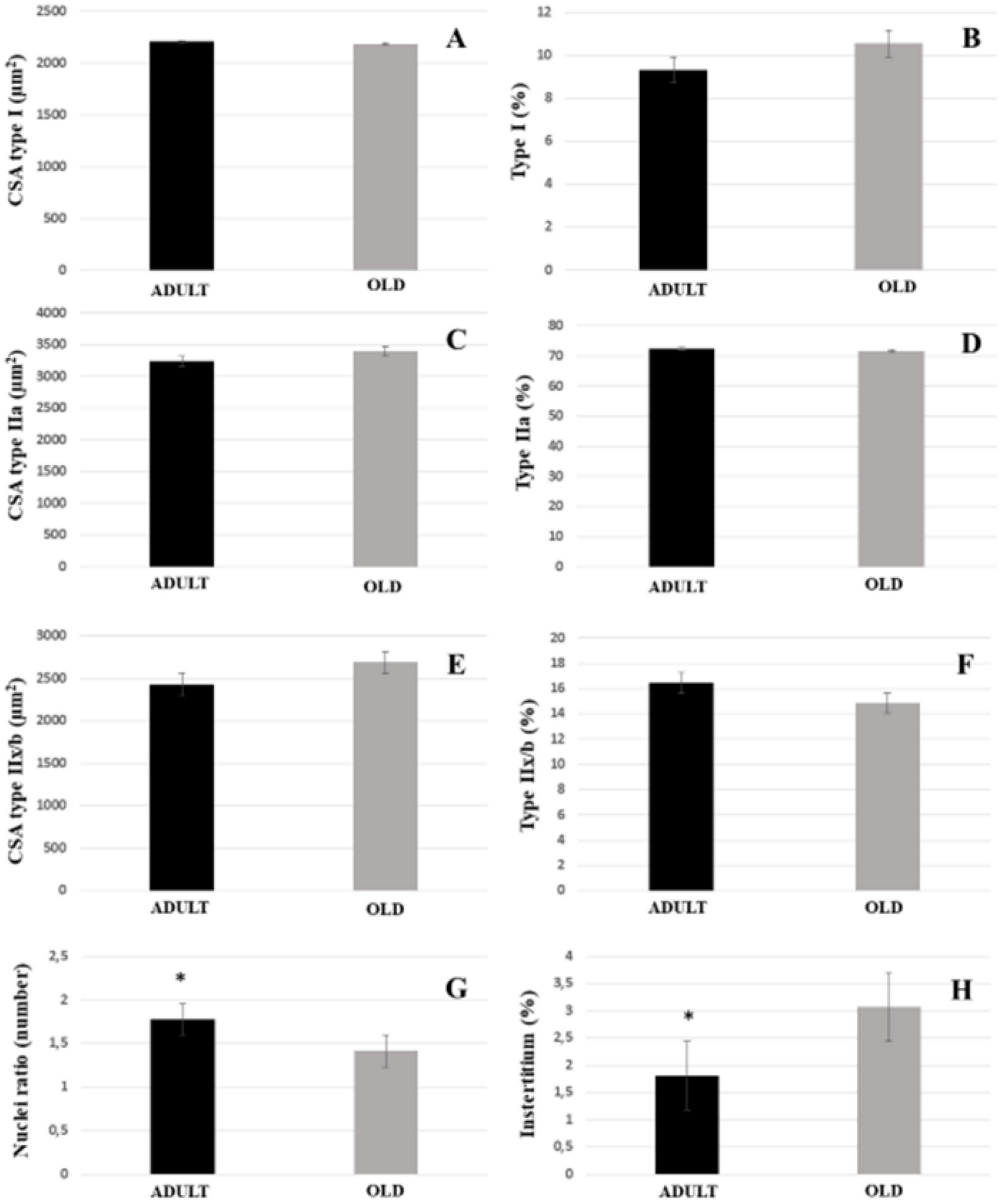
Morphoquantitative parameters of plantaris muscle from adult (10 months-old) and old (24 months-old) Wistar rats. Cross-sectional area of type I **(A)**, IIa **(C)**, and IIx/b **(E)**. Stereology of type I **(B)**, IIa **(D)**, IIx/b **(F)**, and interstitium **(H)**. Nuclei ratio **(G)** was calculated dividing number of nuclei in each field by total number of muscle fibers. ^*^Represents a significant difference between the groups (*p* < 0.05).

For both soleus and plantaris, the interstitial volume density increased significantly in the OLD group compared to the ADULT (*p* < 0.05). The nuclei ratio of OLD group was statistically lower than ADULT (*p* < 0.05). Other outcomes were not statistically different (Figures 14, 15).

#### Ladder climbing test

**Figure 16:**
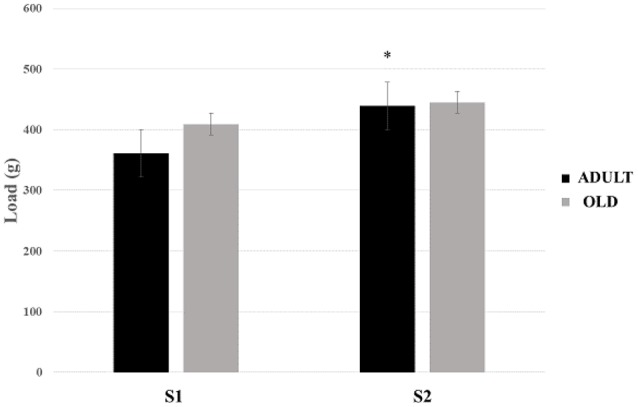
Maximum load-carrying test (MLCT) of sessions 1 (S1) and 2 (S2) from groups ADULT (black bars) and OLD (gray bars). ^*^Indicates statistical trend (*p* = 0.059) between S1 and S2 for ADULT group.

No statistical difference was found between groups for loaded tests (Figure 16). There was a tendency to increase the load carried between S1 and S2 in the rats of the ADULT group (*p* = 0.059).

## Discussion

Here, we demonstrated an enlargement of the area and diameter of the myelinated fibers, in conjunction with a reduction in the amount and space occupied by these nerve fibers. Increasing the cross-sectional area of myelinated fibers might be a way of compensating for the decrease in their amount, and consequently of the space occupied. Also, through the qualitative stereological analysis it was clear that there is a reduction in the amount of myelinated fibers of normal morphology caused by the aging process. Unfortunately, the number of evidence evaluating the tibial nerve of Wistar rats is limited. Thus, we discourse our results from the published evidence, whose investigations have studied other nerves and/or lineages. Corroborating our results, Kanda and Hashizume (1998) and Hashizume and Kanda (1995) demonstrated that aged rats had a significant increase in the diameter of myelinated axons of the medial and ulnar gastrocnemius nerves of Fisher 344 rats. However, Samorajski and Rolsten (1975) indicated that the tibial nerve of C57BL/10 mice shows no reduction of the cross-sectional area nor the number of nerve fibers at 24 months of age. Further ahead, in a very well controlled study, Ceballos et al. (1999) studied the tibial nerve of female Swiss mice between 6 and 33 months of age, describing variability of effects on different MF sizes throughout the aging process. Ugrenovic et al. (2016) found a decrease in the percentage of MF with larger diameters along the advancing age. This data was accompanied by an increase in the percentage of MF of smaller caliber. Such divergence may be present due to the different ages analyzed or the portion of the nerve. Thus, Jeronimo et al. (2008) evaluated the proximal and distal portions of the sural nerve of both right and left limbs of Wistar rats. Although they did not find changes in MF size, myelinated axon area and diameter were significantly reduced in the distal segment of the older age group. Sakita et al. (2016) demonstrated that distal tibial nerve from aged Wistar rats undergo diminished fiber diameter, myelin thickness, axon diameter, myelin perimeter, and axon perimeter. It is clear to this point that there is great divergence in the literature as to what to expect from the morphological alterations in the peripheral nerves, induced by the aging process in the most different strains of animals. This fact indicates that any peripheral nerve can undergo changes in its morphology, ranging from changes in their area and diameter to the quantity and quality of each peripheral nerve cell. It is also possible to see the effect of aging on the structure of the motoneurons (Hashizume and Kanda, 1995). According to Deschenes et al. (2010), NMJ must present changes that lead to a reduction in MF quantity. As this may happen, distal portions shall undergo changes of which might induce motorneurons loss. Also, changes in myelin sheath morphology and SC number can impact in MF quality and quantity (Hinman et al., 2006; Shokouhi et al., 2008; Shen et al., 2011; Sakita et al., 2016).

The myelin sheath is a fundamental component for the electrical transmission in healthy nerves (Melcangi et al., 2003). In the present study, we did not find any variation in the thickness of the myelin sheath between the groups. Our data are corroborated by several other studies in the literature (Hashizume and Kanda, 1995; Jeronimo et al., 2008; Shen et al., 2011). However, Ceballos et al. (1999) verified a significant variation in tibial nerve myelin sheath thickness, without any subsequent change in the G ratio. Knowing that the portion of the nerve analyzed may influence the results investigated, Sakita et al. (2016) demonstrated that both myelin sheath thickness and perimeter of the distal portions of the nerves are significantly lower in aged group. Thus, it is evidenced that aging may initially affect the distal portion of peripheral nerves before proceeding toward to the cell body of the α-motoneuron.

The G ratio is a myelinization index of the nerve fibers and values between 0.6 and 0.7 are best suited for good and maximum MF conduction velocity (Rushton, 1951). Nakayama et al. (1998) mentioned that there is no reduction in the conduction velocities of action potentials in MF. Here, we found that G ration was unchanged. Our results are similar to those found by Sakita et al. (2016), however different from others. Jeronimo et al. (2008) found a significant reduction of the G ratio at the age of 24 months. It is noteworthy to cite that in all the studies presented, different nerves were analyzed.

Interesting variations in the peripheral neuronal structure of aged samples can be found in the literature (Ceballos et al., 1999). It has been reported that MF suffers more than unmyelinated fibers during aging (Sato et al., 1985; Hinman et al., 2006; Shen et al., 2011; Sakita et al., 2016). This is explained by the great variability of myelinic alterations found during the advancing age (Ceballos et al., 1999; Ugrenovic et al., 2016). Ceballos et al. (1999) reported a significant reduction of 50% of UA area with advancing age. Sato et al. (1985) demonstrated that while there is a reduction in the electrical impulse conduction velocity of myelinated fibers, the same result was not found for unmyelinated fibers. The reason for this may lie in the fact that the major changes induced by aging occur in the quality and morphology of the myelin sheath. Soltanpour et al. (2012) investigated peripheral nerve through a very interesting study comparing light and electron microscope techniques. In light microscope, numbers of myelinated nerve fibers, the mean entire nerve perimeters, areas and diameters were not different between the young and aged groups. In electron microscope, numbers of myelinated axons, Schwann cell nuclei and mean G ratios were not significantly different between ages. However, myelinated fiber diameters, myelin sheath thickness, myelinated axon diameters, and mean G ratio were significantly different. Clearly, the mode of analysis may influence the results and induce a different interpretation of nerve changes. Here, stereological analysis only showed difference in total number of normal myelinic fibers between ages. Intriguing, myelinic alterations did not vary between groups. This fact can also be explained by the absence of change at SC number found here. Longitudinally, Ceballos et al. (1999) showed that SC number increased from 12 to 22 months of age. Increased SC number could be an early form to compensated the reduction of MF at later ages. As many other parameters shown here, myelin abnormalities may also take longer time to occur in the Wistar rats lineage.

Striking NMJ denervation may occur without loss of α-motorneurons (Chai et al., 2011). Thus, postsynaptic compartment suffers morphological changes over advancing age (Jang and Van Remmen, 2011; Li et al., 2011; Cheng et al., 2013; Gonzalez-Freire et al., 2014; Rudolf et al., 2014). Here, we found NMJ enlargement at both skeletal muscle types. However, divergent results from ours might be found in the literature. Deschenes et al. (2007) failed to show any changes at early aged SL postsynaptic sites of Fisher 344 rats. Even at ages similar to ours, SL and PL postsynaptic region may not change (Deschenes et al., 2012, 2016). Interesting, for the same rodent ages and lineage, significant changes at end-plate can be show. According to Deschenes et al. (2010), there is no change in the perimeter of NMJ from both type I and II myofibers of soleus muscle, while there is a significant increase in the end-plate of the plantaris muscle. Further, Deschenes et al. (2011) demonstrated that slow contraction myofibers will suffer more changes across age. Yet, Deschenes et al. (2012) found a significant reduction of the stained area and percentage of dispersion in the soleus aged NMJ. Northless, Deschenes et al. (2015) demonstrated an increase over stained and total area, as well as the percentage of dispersion in the age group. Recently, Deschenes et al. (2016) presented a significant increase in the postsynaptic total NMJ perimeter. Li et al. (2011) explained that the dispersion of the end-plate is a result of its fragmentation, caused by an imbalance between the processes of degeneration and regeneration. In our study, we found increased dispersion only in the soleus muscle end-plate. According to Deschenes et al. (2010), the fragmentation of NMJ precedes the onset of sarcopenia and may impact qualitative alterations in the neuromuscular system (Tamaki et al., 2014). There are several mechanisms that may be involved in this regulatory loss. Ibebunjo et al. (2012) demonstrated that a reduction in the mitochondrial function directs these tissues to sarcopenia. A stable state should also be maintained between protein synthesis and degradation at the end-plate area. Drey et al. (2013) suggest that the C-terminal fragment of agrin may be a potential marker for sarcopenia and Hettwer et al. (2013) confirmed that sarcopenia is an agrina-dependent process. According to MacDonald et al. (2017), loss of synaptic homeostasis and plasticity are involved with decreased transcription factor only growth response (Egr-1) and increased agrin. These alterations may lead to decreased movement and limb muscle strength. NMJ instability may leave muscle fibers more susceptible to damage through dystrophin loss. In order to compensate reduction of dystrophin protein, aged muscles might increase α-sarcoglycan, syntrophin, sarcospan, laminin, β1-integrin, desmuslin, and the Z-line proteins α-actinin and desmin (Hughes et al., 2016; Lee et al., 2017). Other mechanisms, such as reduction of tyrosine kinase receptor (TrkB), myogenic regulation factor 4 (MRF-4), insulin-like growth factor- 1 (IGF-1), and reactive oxygen species (ROS) augment may also be involved in NMJ fragmentation (Krause Neto and Gama, 2013; Gonzalez-Freire et al., 2014).

Desaki (2008) indicates that there is an interesting difference between structural changes in muscle fibers during aging, and it may be associated with a reduction in the number of muscle fibers or their size. Also, data show that glycolytic fibers might decrease in size, but not in number, while oxidative fibers decrease in number but not in size (Tauchi et al., 1971). In our study, we demonstrated that there was no significant change in both morphometry and stereology of muscle fibers type I, IIa, or IIx/b of both soleus and plantaris. Corroborating our results, Deschenes et al. (2010) demonstrated maintenance of muscle architecture. However, significant atrophy of type I myofibers of the soleus in the age group was found by Deschenes et al. (2011). Here, plantaris muscle morphometry indicated a significant reduction of type IIa myofibers and an increase in the total proportion of type I. Deschenes et al. (2012) found a significant reduction of all types of muscle fibers, with an increase in the proportion of type I in the soleus. Deschenes et al. (2013a) reported a decrease in the cross-sectional area of type II fibers, with an increase in the proportion of type I fibers and reduction of type II in the soleus muscle, while in the plantaris muscle had an increase in the area of myofibers I and IIa, with reduction of IIb. In 2015, Deschenes et al. found a significant increase in the proportion of type I fibers and plantaris muscle type II enlargement, without any change in the cross-sectional area of these fibers. Recently, Deschenes et al. (2016) demonstrated a reduction in the area of the type I and IIa fibers, with an increase in the proportion and occupied area of the plantaris muscle type IIx fibers. The divergence over studies might be influenced by the variation of the lineages and ages investigated. Chan and Head (2010) quoted that although rodents demonstrate a reduction of muscle mass similar to humans during aging, these reductions may take longer to present.

Remodeling of end-plate morphology may lead to functional changes in mechanical properties such as less efficient contraction, impairment activation of agonists muscles, decreased repair capacity by SC, reduced capacity of motoneurons to reinnervate and sprout, less calcium release, ACh receptors dispersion, and increased Tau protein (Gonzalez-Freire et al., 2014; Yin et al., 2017). Degens and Alway (2003) reported reduced plantaris muscle mass and maximal tetanic force between 9 and 26 months of age. According to Pannérec et al. (2016) forelimbs are resistant to sarcopenia while hindlimbs are impact by age-induced muscle alterations. Regional susceptibility to sarcopenia is dependent on NMJ fragmentation, loss of innervation, reduced excitability and dysregulation of sterol metabolism (Pannérec et al., 2016). In an interesting work, Tamaki et al. (2014) reported that Wistar rats fast-type plantaris muscles may show typical signs of sarcopenia at very old age (30 months-old) such as: reduction of shortening and relaxing velocity of twitch, decline of muscle tenderness, impaired recruitment of MU at higher stimulation frequencies, and easy fatigability in the NMJ. Despite these changes, here Wistar rats did not undergo any changes in skeletal muscle size and composition (only significant increase of the interstitium was demonstrated) or strength-bearing capacity, as measured by the ladder climbing test.

The sarcopenia syndrome represents a risk for falls and loss of physical independence. The reduction of functional capacity in the elderly may be related to supraspinal and spinal factors (Kanda and Hashizume, 1998; Tamaki et al., 2014; Cyran et al., 2015; Godde and Voelcker-Rehage, 2017; Karmali et al., 2017). The decline in the amount of physical effort seen during aging impacts negatively on the day-to-day coordinative and cognitive tasks (Godde and Voelcker-Rehage, 2017). At more medullary levels, a qualitative change begins before typical sarcopenia (Tamaki et al., 2014). Functional alterations induced by aging in the PNS appear to be dependent on the deterioration of larger nerve fibers (Verdú et al., 1996). However, peripheral nerves with different functions (autonomic vs. somatic) present different adjustments during aging (Sato et al., 1985; Nakayama et al., 1998). In somatic nerves, the reduction in the number of motoneurons seems to be compensated by adjustments in the peripheral nerve (Kanda and Hashizume, 1998). Adaptations in the morphology of nerve fibers are related to their electrophysiological properties (Shen et al., 2011; Pousinha et al., 2015; Pannérec et al., 2016). However, the innervation of the sciatic nerve in its target muscles does not change during the advancing age and has a positive correlation between the amplitude of the compound muscle action potentials and the amount of myelin sheath lamellae (Shen et al., 2011). According to Sato et al. (1985), the maximum conduction velocity in the myelinated fibers is reduced only at very advanced ages (between 24 and 30 months). Recently, Pannérec et al. (2016) found that regional susceptibility of sarcopenia is dependent on NMJ fragmentation, loss of motor neuronal innervation and less excitability. Through a very well controlled study, the authors demonstrated that forelimb muscles are more resistant to sarcopenia due to the smaller amount of genes regulated by aging. In all, the triceps brachial muscle is regulated by 196 genes at more advanced age, while more than 6,000 genes regulate the sarcopenic state of the gastrocnemius muscle (Pannérec et al., 2016). Furthermore, hindlimb muscles may present a kinetics of atrophy similar to that pronounced between fast-contracting fibers, however, by mechanisms independent of the muscular contractile phenotype (Pannérec et al., 2016). These alterations have consequences such as lower number of motoneurons, lower compound muscle action potential, greater fragmentation of NMJs, loss of muscle fibers, and consequently lower gait speed and stride length (Pannérec et al., 2016). Moreover, sarcopenia leads to a lower maximal tetanic force, lower resistance to fatigue, impairment of recruitment of MUs, and rapid fatigability of NMJs (Degens and Alway, 2003; Deschenes, 2011; Tamaki et al., 2014). Possibly here, the absence of changes demonstrated in the muscle morphology and ladder climbing test, could be explained by the adaptation of nerve fibers and NMJ not allowing muscle fibers to be denervated. Yet, even though skeletal muscles may present mechanical changes of age, voluntary testing similarly done by humans in daily tasks, may not be impacted. For last, Cheng et al. (2013) suggested that beginning physical activities at middle-age can prevent much of the age-associated loss of nerve terminals regularly show in many studies. Despite all the functional changes mentioned above, it is absent in the literature study that aimed to evaluate the voluntary functional capacity of rodents in efforts that depend on muscular strength. In a single study, Pannérec et al. (2016) demonstrated that changes in gait speed and stride length of 20-month-old Wistar rats occurred along with ultrastructural and molecular changes. This fact strengthens our results, whose ladder climbing load test did not present significant difference between both ages. Clearly, compensatory changes in the neuromuscular system prevented sarcopenia and decrement of the ability to climb ladder carrying weight.

In conclusion, the present study demonstrated that the aging process induces changes in the peripheral nerve and postsynaptic site without any change in skeletal muscles and weight-bearing capacity of Wistar rats until 24 months of age.

## Author contributions

WK: Prepared and analyzed the material and wrote the final version of the article; WS: Matched the material and participated in the discussions on the assembly of the manuscript; AC, RdS, and CA: Participated in the discussions on the assembly of the manuscript; EG: Guided the entire process and reviewed the final version of the manuscript.

### Conflict of interest statement

The authors declare that the research was conducted in the absence of any commercial or financial relationships that could be construed as a potential conflict of interest.
